# Trends in emergency department visits due to back pain and spine surgeries during the COVID-19 pandemic in Finland

**DOI:** 10.1097/MD.0000000000029496

**Published:** 2022-06-10

**Authors:** Saara Jäntti, Ville Ponkilainen, Heikki Mäntymäki, Mikko Uimonen, Ilari Kuitunen, Ville M. Mattila

**Affiliations:** a Faculty of Medicine and Health Technology, Tampere University, Tampere, Finland; b Department of Surgery, Central Finland Hospital Nova, Hoitajantie 3, Jyväskylä, Finland; c Department of Orthopaedics and Traumatology, Tampere University Hospital, Teiskontie 35, PL2000, Tampere, Finland; d Mikkeli Central Hospital, Porrassalmenkatu 35-37, Mikkeli, Finland; e University of Eastern Finland, School of Medicine, Yliopistonranta 1, Kuopio, Finland.

**Keywords:** back pain, COVID-19, emergency, spine surgery, urgent surgery

## Abstract

We aim to report the incidences of ED visits due to back pain, hospitalizations, and urgent spine surgeries during the first and second waves of COVID-19 in Finland. The number of emergency department visits and hospitalizations due to back pain as well as urgent spine surgeries in the adult population was collected from hospital discharge registers for the years 2017 through 2019 (reference years) and 2020.

This study was conducted at three large Finnish hospitals. The monthly incidence with 95% confidence intervals (CI) of emergency department visits and hospitalizations due to back pain and spine surgeries in the three participating hospitals were calculated and compared by incidence rate ratios (IRR).

Visits to ED due to back pain decreased during the pandemic. The incidence of ED visits due to back pain was similar in February (IRR 0.95, CI: 0.82-1.10), but a decrease was seen after lockdown began (March IRR 0.67, CI: 0.57-0.78; April IRR 0.65, CI: 0.56-0.76) compared to the reference years. A second decrease in visits was seen after regional restrictions were implemented in October (IRR 0.88, CI: 0.76-1.02). The most common diagnoses were non-specific back pain, lumbar disk herniation, and back contusion. Incidence of non-specific back pain decreased during the lockdown (March IRR 0.65, CI: 0.55-0.78) and regional restrictions (October IRR 0.83, CI: 0.70-0.98), whereas the rates of other diagnoses remained unchanged, and incidences of hospitalizations and urgent spine surgeries remained stable.

A clear decrease in ED visits due to back pain was seen during the first and second waves of the pandemic. This decrease was mainly the result of patients with non-specific back pain avoiding visits to the ED. The incidence of specific back pain, hospitalizations, and urgent spine surgeries remained unchanged during the pandemic.

## 1 Introduction

The rapid spread of the Severe Acute Respiratory Syndrome Coronavirus-2 (SARS-CoV-2), more commonly known as COVID-19, has impacted health care across the world.^[1]^ In Finland, the Government declared a state of emergency and national lockdown in March 2020 due to the coronavirus outbreak. Thereafter, guidelines emphasizing social distancing as a necessary strategy to reduce viral spread resulted in a rapid decrease in the number of COVID-19 cases. At the time of the lockdown in March, schools were closed, gatherings were limited to up to 10 persons, and public indoor premises were closed. In addition, traveling was restricted from March to June. Schools were reopened in mid-May.^[2]^

The second wave of COVID-19 infections began in Finland in the fall of 2020. However, no state of emergency was declared. Instead, targeted regional restrictions were implemented, where necessary. Furthermore, the Finnish Institute for Health and Welfare (THL) recommended the wearing of facial masks for individuals 15 years and older in situations where keeping a safe distance was not possible. The epidemiologic situation was divided into three levels: base level, acceleration level, and spreading level.

Back pain is a common complaint in the adult population and also a major cause of emergency department (ED) visits.^[3–5]^ During the first wave of the COVID-19 pandemic, however, a clear reduction in the number of ED visits due to lower back pain was reported.^[6,7]^

The COVID-19 pandemic also decreased the volume of spine surgeries performed,^[8,9]^ with the exception of the number of surgical decompressions performed for cauda equina syndrome, which did not decrease during the first wave of the COVID-19 pandemic.^[10]^

The aim of this study is to evaluate the incidence of ED visits and hospitalizations due to back pain and the rates of urgent spine surgery during the first and second waves of the COVID-19 pandemic in Finland.

## 2 Methods

This study was conducted at three large Finnish public hospitals. Tampere University Hospital (tertiary level unit), Mikkeli Central Hospital (secondary level unit with integrated primary care ED), and Central Finland Hospital (secondary level unit with integrated primary care ED) cover a catchment area of approximately 900,00 inhabitants.^[11]^ The number of emergency department visits and hospitalizations due to back pain as well as emergency spine surgery in the adult population (age 18 or older) was collected from patient information systems using the International Classification of Diseases 10th Revision (ICD-10)^[12]^ diagnostic codes for back pain. As our aim was to include all emergency department visits and operations due to back pain, we gathered all patients with spine specific ICD-10 codes (Table 1). The visits due to back pain were classified into the following groups: non-specific back pain, lumbar disk herniation, back contusion, lumbar spine fracture, spinal stenosis, and thoracic spine fracture. All patients who were admitted to the participating hospitals with back pain in the year 2020 and the years 2017 through 2019 (reference years) were included. Ethical committee approval was not necessary for this study since this is a retrospective study.

**Table 1:** The classification of ED visits due to back pain and spine surgeries according to diagnostic and procedure codes.

ED visits due to back pain		Spine surgeries	
**Non-specific back pain**		**Disc surgery**	
M51.9	Unspecified intervertebral disc disorder	ABC16	Excision of lumbar intervertebral disc displacement
M53.8	Other specified dorsopathies	ABC23	Open discectomy of thoracic spine
M53.9	Dorsopathy, unspecified	ABC26	Open discectomy of lumbar spine
M54.0	Panniculitis of back	**Decompression**	
M54.3	Ischias	ABC33	Decompression of thoracic nerve roots
M54.4	Lumbago with sciatica	ABC36	Decompression of lumbar nerve roots
M54.5	Low back pain	ABC53	Decompression of thoracic spinal canal and nerve roots
M54.6	Pain in thoracic spine	ABC56	Decompression of lumbar spinal canal and nerve roots
M54.8	Other dorsalgia	**Fracture**	
M54.9	Dorsalgia, unspecified	NAJ22	External fixation of fracture of thoracic spine
		NAJ30	Internal fixation of fracture of cervical spine
**Lumbar disc herniation**		NAJ32	Internal fixation of fracture of thoracic spine
M51.0	Intervertebral disc disorders with myelopathy	**Fusion**	
M51.1	Disc disorders with radiculopathy	NAG52	Interbody fusion of thoracic spine with external fixation
		NAG53	Interbody fusion of thoraco-lumbar spine with external fixation
**Back contusion**		NAG57	Interbody fusion of spine with external fixation
S23.0	Traumatic rupture of thoracic intervertebral disc	NAG62	Interlaminary fusion of thoracic spine without fixation
S23.3	Sprain of ligaments of thoracic spine	NAG63	Interlaminary fusion of thoraco-lumbar spine without fixation
S30.0	Contusion of lower back and pelvis	NAG66	Interlaminary fusion of lumbo-sacral spine without fixation
**Lumbar spine fracture**		NAG99	Other excision, reconstruction, or fusion
S32.0	Fracture of lumbar vertebra	**Other**	
S32.7	Multiple fracture of lumbar vertebra	NAR00	Incomplete excision of soft tissue tumor of spine
**Spinal stenosis**		NAR99	Other operation for tumor of spine
M48.0	Spinal stenosis	NAK10	Partial or total excision of vertebra
M47.2	Spondylosis with radiculopathy	NAK99	Other operation of vertebra
**Thoracic spine fracture**		NAS99	Other operation for infection of tendon, joint, disk or bone of spine
S22.0	Fracture of thoracic vertebra	NAW00	Reoperations on spine and neck
S22.1	Multiple fracture of thoracic vertebra	NAW10	Reoperations on spine and neck
		NAW99	Reoperations on spine and neck

ED = emergency department.

Information on and the number of urgent spine surgeries (delay less than 14 days) during the year 2020 and the reference years were retrospectively collected and confirmed from the electronic medical record systems of the participating hospitals using NOMESCO Classification of Surgical Procedures (NCSP)^[13]^ procedure codes (Finnish version). Spine surgeries were classified into the following groups: disk surgery, decompression, fracture, fusion, and other (Table 1).

Hospitalizations were classified into two groups: patients with non-specific back pain and patients with other back pain including all other reasons. Monthly incidences with 95% CI were counted per 100,00 person-months by Poisson exact method and compared by incidence rate ratios (IRR). The analyses and figures were performed using R version 3.6.2 (R Foundation for Statistical Computing, Vienna, Austria).

## 3 Results

A total of 4310 visits due to back pain occurred during the year 2020, and 376 urgent spine surgeries were performed during the same period. The mean age of patients visiting ED unit due to back pain was 54 years and 55% of them were women. For urgent spine surgeries, the mean age of patients was 56 years and 57% of them were men. During the reference years (2017–2019), the mean number of visits due to back pain was 4884 and 307 for urgent spine surgeries.

**Figure 1:**
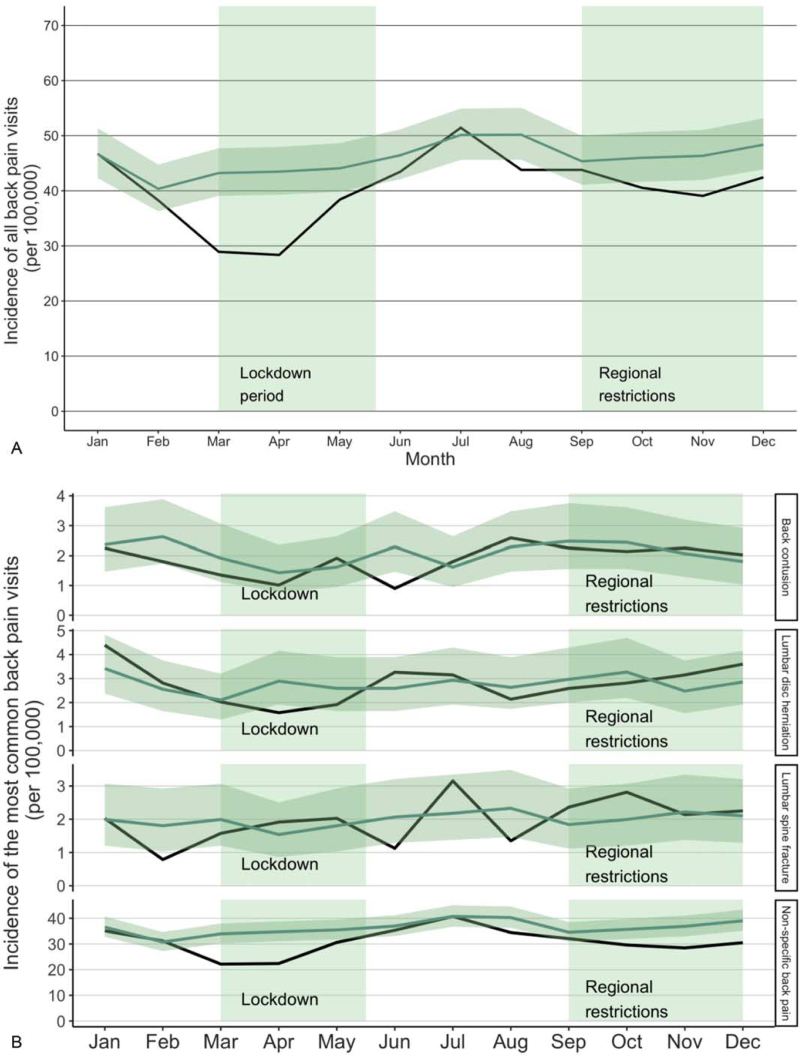
Incidence of all visits due to back pain (A) and the most common visits due to back pain (B) during the COVID-19 pandemic. The dark line illustrates the incidence during the study period (2020) and the lighter line illustrates the mean of incidences in the reference years (2017–2019) with confidence intervals.

The incidence of ED visits due to back pain decreased during the first and second waves of COVID-19 pandemic (Fig. 1A). When compared to the reference years, the incidence of visits due to back pain in February 2020 was similar to previous years (IRR 0.95, CI: 0.82–1.10), but a notable decrease was seen in March (IRR 0.67, CI: 0.57–0.78). The incidence of back pain visits rebounded to its previous level after the lockdown period ended in June, but again showed a slight decrease when regional restrictions were implemented in October (IRR 0.88, CI: 0.76–1.02), with the largest decrease in November (IRR 0.84, CI: 0.73–0.97).

The most common reasons for visits to the ED due to back pain were non-specific back pain, lumbar disk herniation, back contusion, and lumbar spine fracture (Fig. 1B). The incidence of non-specific back pain decreased both during the lockdown and during the regional restrictions. A decrease was first seen from February (IRR 1.01, CI: 0.86–1.20) to March (IRR 0.65, CI: 0.55–0.78). The incidence of non-specific back pain remained at the same level as in the reference years between May and September, but a further decrease was seen in October (IRR 0.83, CI: 0.70–0.98). However, the incidence of lumbar disk herniations, back contusions, and lumbar spine fractures was similar when compared to the reference years.

**Figure 2:**
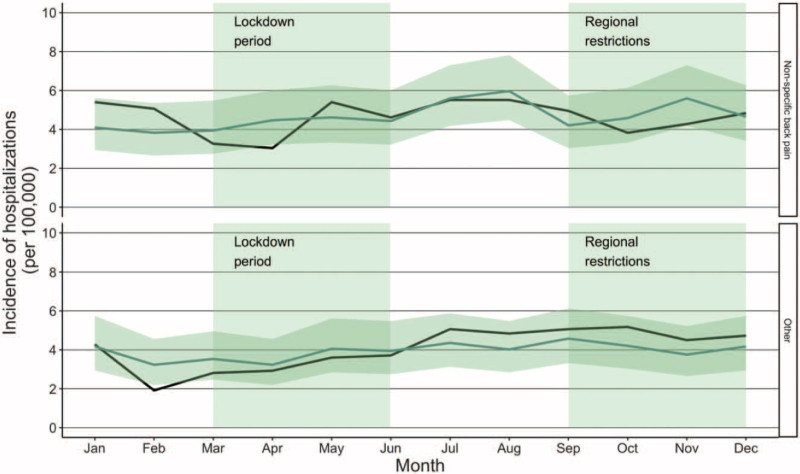
Incidence of hospitalizations during the COVID-19 pandemic. The dark line illustrates the incidence during the study period (2020) and the lighter line illustrates the mean of incidences in the reference years (2017–2019) with confidence intervals.

The incidence of hospitalizations remained stable during the COVID-19 pandemic (Fig. 2). The IRR of hospitalizations in patients with non-specific back pain was 0.68 (CI: 0.42–1.11) during the lockdown in April and 0.83 (CI: 0.53–1.32) during the regional restrictions in October. The IRR of hospitalizations in patients with other back pain was 0.89 (CI: 0.55–1.43) in May and 1.23 (CI: 0.80–1.89) in October.

**Figure 3:**
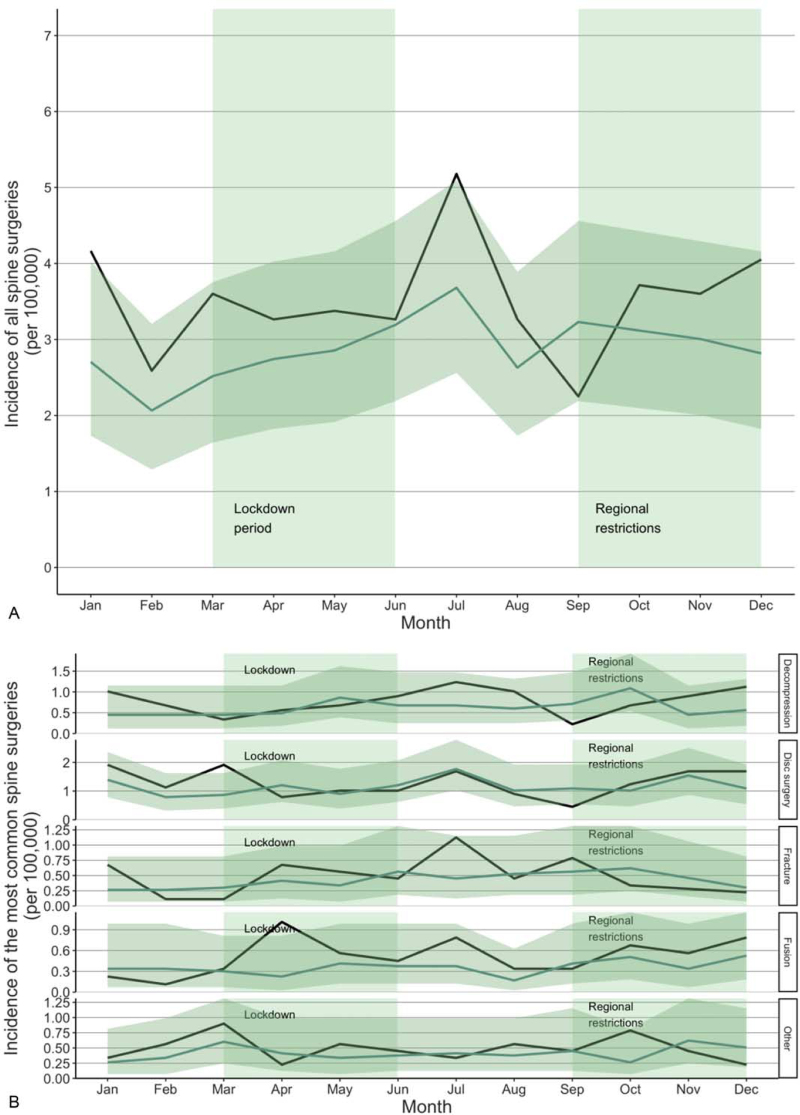
Incidence of all spine surgeries (A) and the most common spine surgeries (B) during the COVID-19 pandemic. The dark line illustrates the incidence during the study period (2020) and the lighter line illustrates the mean of incidences in the reference years (2017–2019) with confidence intervals.

The incidence of all urgent spine surgeries remained at the same level in comparison to the reference years (Fig. 3A). In the middle of the lockdown in May, the IRR of all spine surgeries was 1.18 (CI: 0.70–2.01). During the regional restrictions, the IRR was at the same level, being 1.19 in October (CI: 0.72–1.97). The most common spine surgeries were disk surgery, decompression, fusion, and fracture (Fig. 3B). The incidence of the most common spine surgeries remained stable during the first and second waves of the COVID-19 pandemic. The IRR of the most common spine surgery, disk surgery, was 1.12 (CI: 0.43–2.91) in May and 1.22 (CI: 0.51–2.95) in October.

## 4 Discussion

The results of this study show that the incidence of visits to the ED due to back pain decreased during the first and second waves of the pandemic. Previous studies investigating the first wave of the COVID-19 pandemic have reported similar findings.^[6,7,14]^ In February and March, ED and ICU personnel were redeployed due to COVID-19, and citizens were advised to avoid all non-necessary health care visits. These steps likely led to a decreased number of visits to EDs due to nonspecific back pain, which may reflect the decreased willingness of patients with minor conditions to visit the ED. Further, the incidence of lumbar disk herniations, back contusions, and lumbar spine fractures were similar when compared to the reference years, indicating that patients with severe back pain visited the ED at a rate similar to that before the pandemic.

Consequently, during the study period, the incidence of hospitalizations and spine surgeries remained stable in comparison to the reference years. This also supports the finding that similar numbers of patients with severe pain and prominent symptoms were admitted to EDs in spite of the COVID-19 pandemic. A similar finding was reported in a previous study that showed that the volume of surgical decompressions performed for cauda equina syndrome did not decrease during the first wave of the COVID-19 pandemic.^[10]^ However, the literature describing the changes in neurosurgical procedures during the COVID-19 pandemic has reported increased surgical cancellation rates and a decline in the number of operations, especially elective operations.^[15–17]^ The stable number of urgent spine surgeries may indicate that cancellations of elective spine surgery have not caused patients on waiting lists for surgery to shift to become emergency patients. It may also be the case that a some of the elective operations may have been reorganized to emergency operations. However, we did not address this issue in our study.

The strengths of our study include the broad data that included all visits and surgeries due to back pain in three large Finnish hospitals during the years 2017–2020. The data were comprehensive, and all patients within the study hospital districts were admitted to the study hospitals. Furthermore, we were able to collect follow-up data from all patients during the first and second waves of the COVID-19 pandemic. Our current study also has some limitations. The ICD-10 diagnostic codes for back pain only include specific codes and do not accurately define symptoms. Therefore, non-specific back pain includes a variety of symptoms. We were not able to use imaging to categorize the patients since we did not have access to imaging data. Even though we collected all the ED visits due to back pain and all spine surgeries from the three large study hospitals, data from those patients who received treatment only in primary health care and were not admitted to hospital are lacking. However, as all patients with severe symptoms are referred to our study hospitals, the missing data might only concern the number of patients with unspecific, non-severe back pain.

In conclusion, the incidence of ED visits due to back pain decreased during the first wave of COVID-19 pandemic. A decrease was also seen during the second wave of pandemic. The decrease in incidence can be explained by patients with non-specific back pain avoiding visits to the ED. The incidence of hospitalizations and urgent spine surgeries did not, however, change during the pandemic. The results of this study can be used when preparing for future pandemics and additionally when planning ED triage for patients with back pain, as severe cases were still treated during the pandemic.

## Author contributions

**Data curation:** Saara Jäntti, Ville Ponkilainen

**Formal analysis:** Saara Jäntti

**Investigation:** Heikki Mäntymäki, Ilari Kuitunen, Saara Jäntti, Ville Mattila, Ville Ponkilainen

**Methodology:** Ilari Kuitunen, Mikko Uimonen, Saara Jäntti, Ville Mattila, Ville Ponkilainen

**Project administration:** Ville Mattila

**Resources:** Saara Jäntti

**Supervision:** Heikki Mäntymäki, Ilari Kuitunen, Ville Mattila, Ville Ponkilainen

**Validation:** Heikki Mäntymäki, Mikko Uimonen

**Visualization:** Saara Jäntti

**Writing – original draft:** Saara Jäntti

**Writing – review & editing:** Heikki Mäntymäki, Ilari Kuitunen, Mikko Uimonen, Ville Mattila, Ville Ponkilainen
